# Bidirectional Mendelian randomization analysis of hypertension, coronary artery disease, and gastric cancer with supplementary clinical data

**DOI:** 10.3389/fcvm.2026.1803696

**Published:** 2026-04-21

**Authors:** Qian Ma, Ya-Fei Kong, Ai-Li Li, Li-Na Song, Yun-Meng Tian

**Affiliations:** 1 Department of Gastroenterology, Binhai New Area Hospital of Traditional Chinese Medicine, Tianjin, China; 2 Shanghai University of Traditional Chinese Medicine, Shanghai, China

**Keywords:** coronary artery disease, gastric cancer, genetic causality, hypertension, Mendelian randomization

## Abstract

**Background:**

The relationship between gastric cancer and cardiovascular traits, including hypertension and coronary artery disease (CAD), remains incompletely understood. Observational studies are prone to confounding and reverse causation, and genetic evidence may help clarify the nature of these associations.

**Methods:**

We conducted a bidirectional two-sample Mendelian randomization (MR) analysis using publicly available genome-wide association study (GWAS) summary statistics to investigate the relationships between gastric cancer, hypertension, and CAD. Multiple MR methods and sensitivity analyses were applied to assess robustness. To provide supplementary clinical context, we additionally conducted a small retrospective clinical analysis of 45 individuals, including gastric cancer cases and non-cancer controls, using logistic regression adjusted for age and sex.

**Results:**

MR analyses showed no evidence that genetic liability to gastric cancer was associated with the risk of hypertension or CAD. In contrast, genetic predisposition to hypertension was inversely associated with gastric cancer risk. These findings were consistent across sensitivity analyses. In the retrospective cohort, hypertension was not significantly associated with gastric cancer risk.

**Conclusions:**

This study provides genetic evidence supporting an inverse association between hypertension liability and gastric cancer risk. However, the supplementary retrospective clinical analysis was limited by its small sample size and did not provide independent validation of the MR findings. Larger observational studies are needed. Further studies are warranted to clarify the underlying biological mechanisms.

## Introduction

Gastric cancer remains one of the most common malignancies worldwide and a leading cause of cancer-related mortality, particularly in East Asian populations (1, 2). Despite advances in screening and treatment, its etiology is multifactorial and incompletely understood, involving genetic, environmental, and metabolic factors. Meanwhile, cardiovascular diseases such as hypertension and coronary artery disease (CAD) are among the most prevalent non-communicable diseases globally and share multiple risk determinants with gastric cancer (3), including inflammation, oxidative stress, obesity, and lifestyle factors such as smoking and diet (4, 5).

Accumulating evidence has suggested potential epidemiological associations between CVDs and cancer, yet the direction and causality of these relationships remain controversial. Several observational studies have reported higher cancer incidence among hypertensive or cardiac patients, whereas others have indicated that certain cardiovascular conditions or medications (e.g., renin–angiotensin system inhibitors) might reduce cancer risk (6–9). However, traditional epidemiological designs are prone to confounding and reverse causality, making it difficult to disentangle whether cardiovascular traits contribute to, or result from, cancer pathogenesis. To overcome these limitations, Mendelian randomization (MR) offers a powerful approach by using genetic variants as instrumental variables (IVs) to infer causality between risk factors and outcomes under the principle of random allele assortment at conception.

In this study, we conducted a bidirectional two-sample MR analysis to comprehensively evaluate the potential causal relationships between gastric cancer, hypertension, and coronary artery disease. We aimed to determine (i) whether genetic liability to gastric cancer causally influences the risk of hypertension or CAD, and (ii) whether genetic predisposition to these cardiovascular traits affects gastric cancer susceptibility. Clarifying these relationships may provide new insights into the shared genetic and biological mechanisms linking cardiovascular and gastrointestinal diseases, with potential implications for risk prediction and therapeutic targeting.

## Methods

### Study design

This study adopted a bidirectional two-sample MR design to evaluate the causal relationships between gastric cancer, hypertension, and CAD.

Forward analyses assessed whether genetic liability to gastric cancer affects cardiovascular traits, whereas reverse analyses examined whether genetic predisposition to hypertension or CAD influences gastric cancer risk.

**Figure 1:**
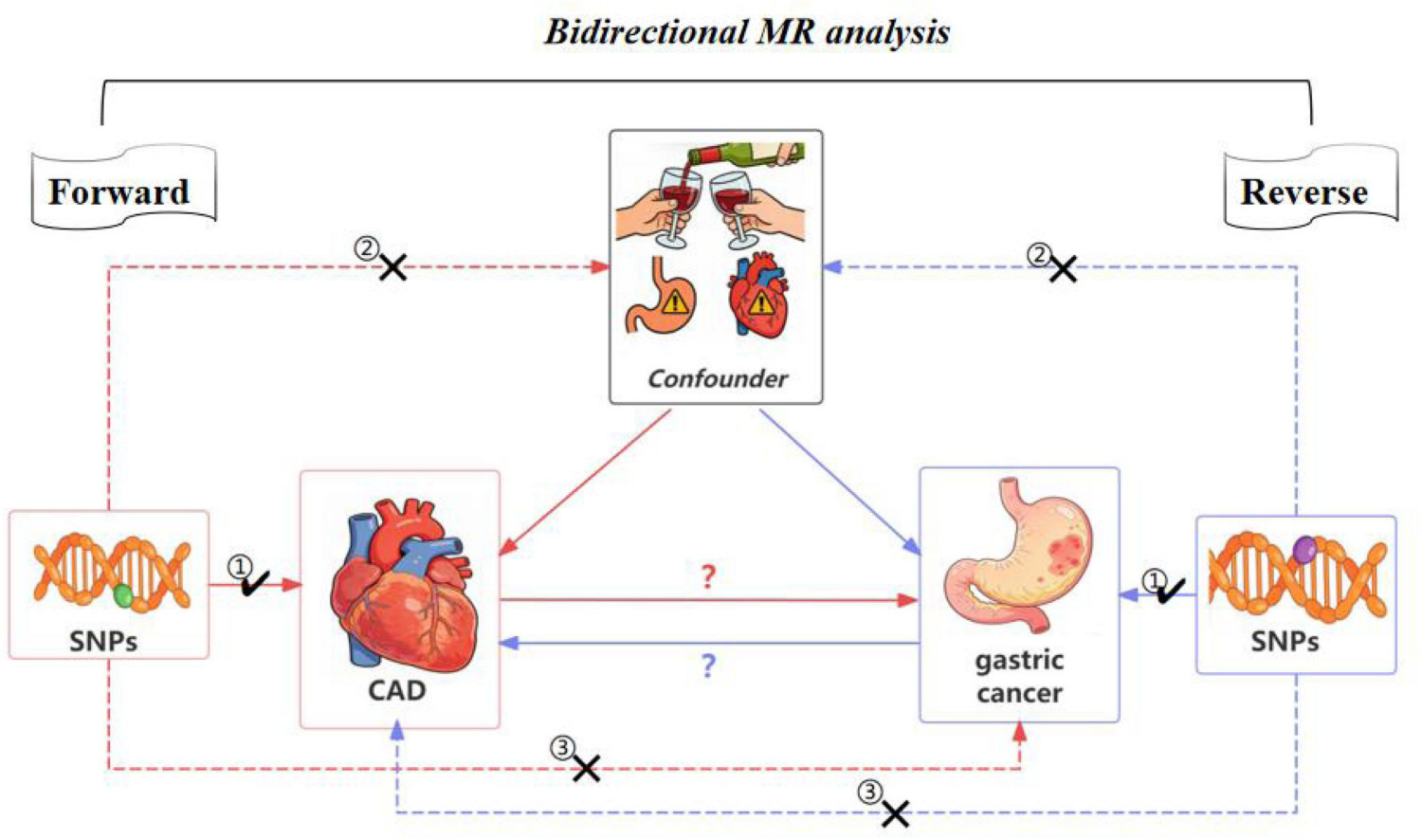
Conceptual framework of the bidirectional MR analysis.

MR analysis relies on three assumptions (Figure 1):

(1) Relevance — the selected genetic variants are strongly associated with the exposure;
(2) Independence — the instruments are not associated with confounders;
(3) Exclusion restriction — the instruments affect the outcome only through the exposure.

In this study, these assumptions were addressed by selecting genome-wide significant and LD-independent SNPs, assessing instrument strength (F > 10), screening variants for confounder associations, and evaluating pleiotropy using MR-Egger, MR-PRESSO, heterogeneity statistics, and leave-one-out analyses.

All analyses used publicly available GWAS summary statistics; therefore, no additional ethical approval was required.

### Data sources and GWAS summary statistics

Summary-level genome-wide association study (GWAS) data for gastric cancer, hypertension, and coronary artery disease (CAD) were obtained from publicly available large-scale datasets through the OpenGWAS platform. The gastric cancer summary statistics were derived from OpenGWAS dataset ebi-a-GCST90018849, including 1,029 cases and 475,087 controls of European ancestry (total *N* = 476,116). The hypertension GWAS summary statistics were obtained from dataset ieu-b-5144, including 133,680 cases and 329,146 controls of European ancestry (total *N* = 462,826). The CAD summary statistics were obtained from dataset ebi-a-GCST90013868, comprising 352,063 participantsof European ancestry.

All datasets were based on the hg19/GRCh37 reference genome build and were restricted to participants of European descent, thereby reducing potential bias due to population stratification in the two-sample Mendelian randomization framework. Detailed phenotype definitions, genotyping procedures, quality-control protocols, and covariate adjustments were as described in the original GWAS publications and corresponding OpenGWAS records. All summary statistics used in this study were publicly available and de-identified; therefore, no additional ethical approval was required for the present analysis.

All analyses were performed using R software (version 4.3.1) with the Two Sample MR (version 0.6.2) and ieugwasr (version 0.1.6) packages.

### Selection of instrumental variables (IVs)

Independent SNPs associated with each exposure at genome-wide significance (*p* < 5 × 10^−^⁸)were selected as IVs. Variants were clumped for linkage disequilibrium (LD r^2^ ≤ 0.001, window = 10 Mb) using PLINK 1.9. Palindromic SNPs with intermediate allele frequencies were excluded. The F-statistic (*β*^2^/SE^2^) was used to assess instrument strength, and all included SNPs had F > 10. After harmonization with each outcome dataset, 7 SNPs for gastric cancer, 164 SNPs for hypertension, and 44 SNPs for CAD were retained for MR analyses.

### Mendelian randomization analyses

The IVW method was used as the primary estimator, complemented by MR-Egger, weighted median, simple mode, and weighted mode methods to evaluate robustness. Bidirectional MR analyses were conducted using the mr() function in the TwoSampleMR R package (v0.6.2). Results were expressed as *β* coefficients, standard errors (SE), and *p* values, representing the causal effect of genetically predicted exposure on the outcome.

### Instrument strength assessment

To ensure instrument validity, we evaluated the strength of all selected genetic variants. Genome-wide significant SNPs (*p* < 5 × 10^−^⁸) were clumped for linkage disequilibrium (LD) at r^2^ < 0.001 using the 1,000 Genomes reference panel. For each exposure, the F-statistic for every SNP was calculated using the formula F = *β*^2^/SE^2^. Across all analyses, the mean F-statistics were substantially greater than the conventional threshold of 10, indicating a low risk of weak-instrument bias.

For the forward MR analysis (gastric cancer → hypertension/CAD), 7 LD-independent SNPs were retained as instruments, all exhibiting strong instrument strength (F > 10). For the reverse MR analyses, 164 SNPs for hypertension and 44 SNPs for CAD were included as exposure instruments, with all variants similarly demonstrating adequate F-statistics above 10. No instrument was removed due to weak strength. These results suggest that the selected genetic variants were unlikely to be affected by substantial weak-instrument bias. However, the limited number of available SNPs for gastric cancer may have reduced statistical power and precision, particularly in the forward MR analyses.

### Statistical power

Statistical power was evaluated using the mRnd online Mendelian randomization power calculator (https://shiny.cnsgenomics.com/mRnd/). Power calculations incorporated the sample size of each GWAS dataset, the number of SNPs included as instruments, and the approximate variance in the exposure explained by the selected genetic variants.

For the forward MR analyses (gastric cancer → hypertension/CAD), the instrument set included 7 genome-wide significant SNPs. Under the sample size of the cardiovascular outcome GWAS and assuming a moderate proportion of explained variance, the analyses yielded >80% power to detect causal effects corresponding to an odds ratio (OR) ≥ 1.20 per standard deviation increase in genetic liability to gastric cancer.

For the reverse MR analyses (hypertension/CAD → gastric cancer), instrument sets consisted of 164 SNPs (hypertension) and 44 SNPs (CAD). Given the substantially larger sample size of the exposure GWAS and the greater variance explained, the reverse analyses demonstrated >90% power to detect modest causal effects equivalent to an OR ≥ 1.10.

These results indicate that the study was adequately powered to detect small-to-moderate causal effects in both directions.

### Confounder screening

To minimize violations of the independence assumption, each candidate SNP was queried in PhenoScanner v2 to identify associations with known confounders such as BMI, smoking, alcohol use, diabetes, lipid levels, socioeconomic factors, and inflammation markers. SNPs associated with confounders at genome-wide significance (*p* < 5 × 10^−^⁸) were excluded. The final instrument sets contained only SNPs without known pleiotropic pathways related to confounding phenotypes.

### Sensitivity and colocalization analyses

Heterogeneity was evaluated using Cochran's *Q* test, and horizontal pleiotropy was assessed by MR-Egger intercept and MR-PRESSO global tests. The leave-one-out approach was used to identify influential variants. Colocalization analyses were performed using the coloc R package to examine whether exposure and outcome traits share the same causal variant within each locus. All statistical tests were two-sided, with *p* < 0.05 considered statistically significant.

## Results

### Forward MR analysis (gastric cancer → hypertension and CAD)

**Figure 2:**
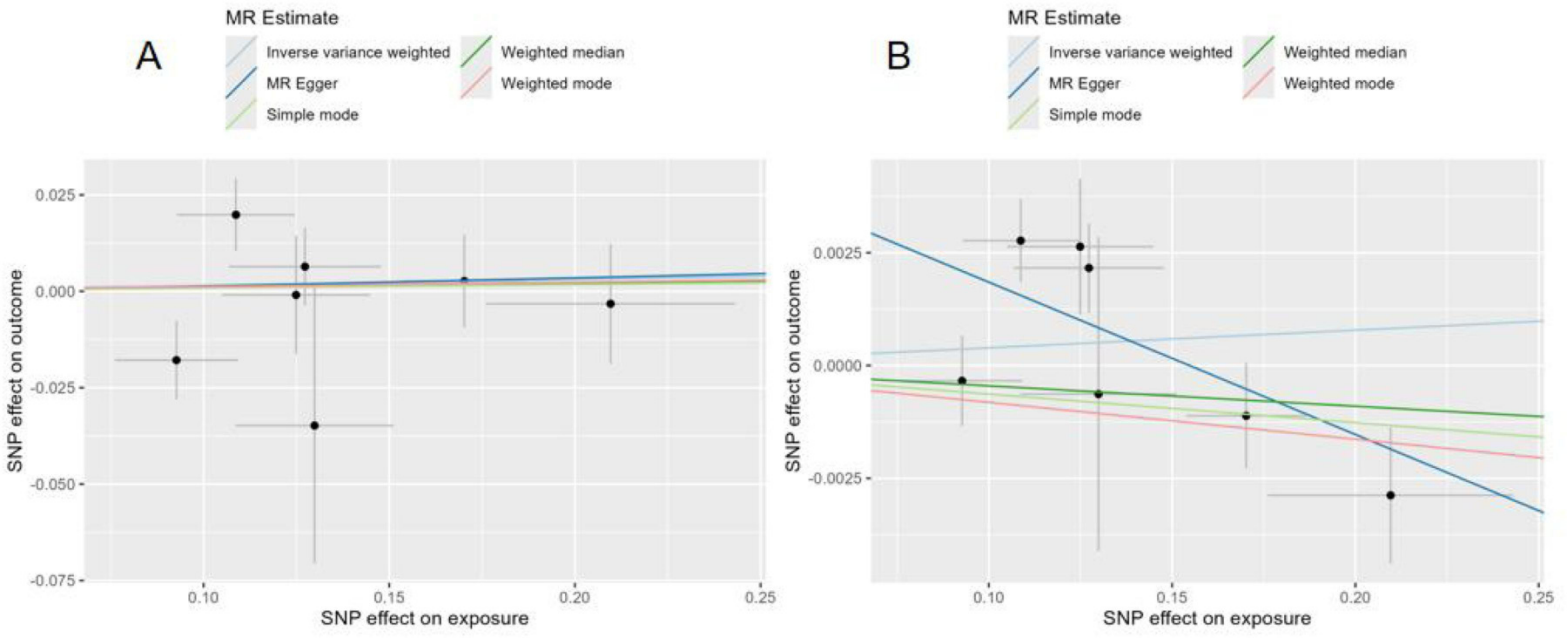
Forward MR analyses of genetically predicted gastric cancer on CAD and hypertension.

In the forward MR analysis, we evaluated the potential causal effects of genetically predicted gastric cancer on the risks of hypertension and CAD (Figure 2). After harmonization, seven independent SNPs were retained as instrumental variables for gastric cancer in each analysis. For hypertension, no evidence of a causal relationship was observed. The IVW method yielded a non-significant association (*β* = 0.0039, SE = 0.0062, *P* = 0.53). Consistent results were obtained using the MR-Egger (*β* = −0.0338, *P* = 0.15), weighted median (*β* = −0.0045, *P* = 0.40), simple mode (*β* = −0.0063, *P* = 0.67), and weighted mode (*β* = −0.0082, *P* = 0.31) approaches, indicating no causal effect of gastric cancer on blood pressure risk. Similarly, for CAD, genetically predicted gastric cancer showed no significant causal effect. The IVW method estimated a null association (*β* = 0.0154, SE = 0.0418, *P* = 0.71), and the direction and magnitude of the effect were consistent across MR-Egger (*β* = 0.0218, *P* = 0.91), weighted median (*β* = 0.0109, *P* = 0.81), simple mode (*β* = 0.0088, *P* = 0.90), and weighted mode (*β* = 0.0118, *P* = 0.83) (Table 1). The overall results suggest that genetically determined susceptibility to gastric cancer does not causally influence the development of hypertension or CAD.

**Table 1:** Forward MR analysis’ results (gastric cancer → hypertension and CAD).

Outcome	MR Method	nsnp	Beta (*β*)	SE	*P* value
Hypertension	IVW	7	0.0039	0.0062	0.5273
	MR-Egger	7	−0.0338	0.0196	0.1453
	Weighted median	7	−0.0045	0.0053	0.3955
	Simple mode	7	−0.0063	0.0142	0.6708
	Weighted mode	7	−0.0082	0.0074	0.3143
CAD	IVW	7	0.0154	0.0418	0.7128
	MR-Egger	7	0.0218	0.1771	0.9067
	Weighted median	7	0.0109	0.0461	0.8135
	Simple mode	7	0.0088	0.0642	0.8957
	Weighted mode	7	0.0118	0.0524	0.8297

### Reverse MR analysis (hypertension and CAD → gastric cancer)

**Figure 3:**
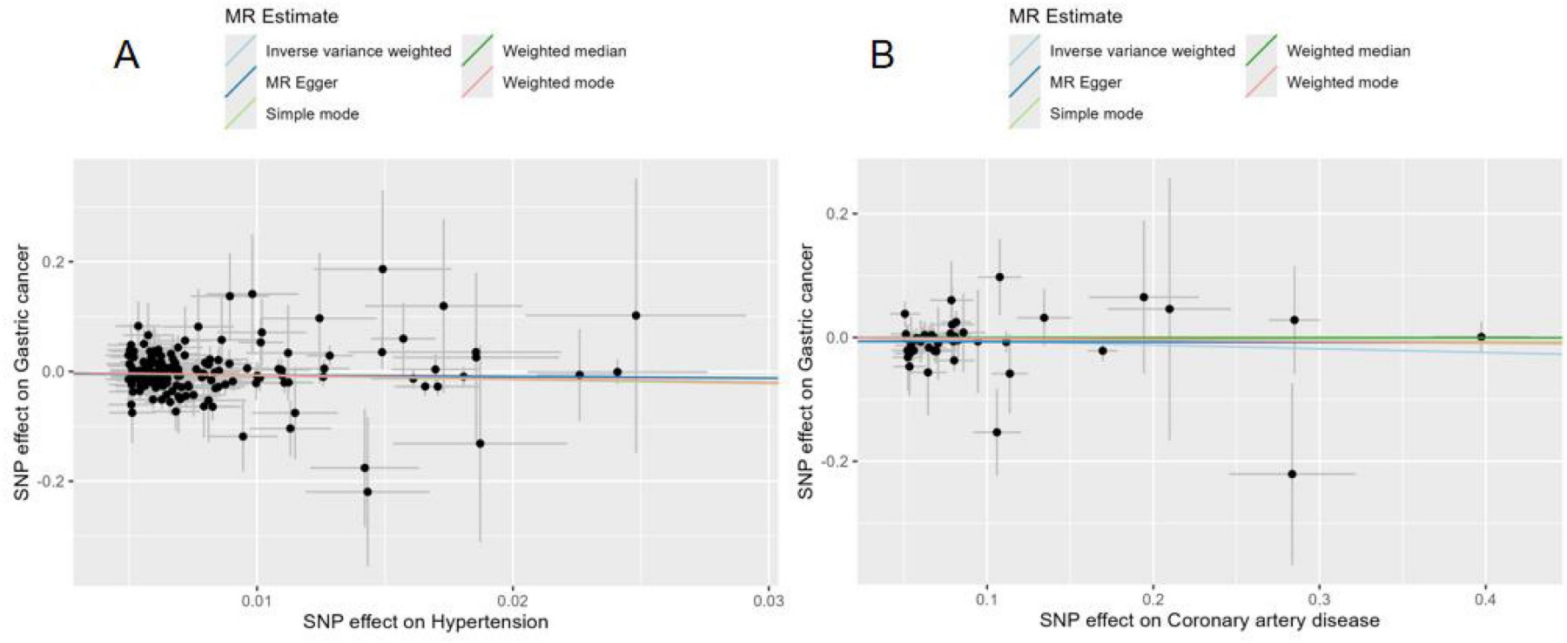
Reverse MR analyses of genetically predicted CAD and hypertension on gastric cancer.

In the reverse MR analysis, we assessed whether genetic predisposition to hypertension or CAD causally influenced the risk of gastric cancer (Figure 3). After harmonization, 164 independent SNPs were retained as instrumental variables for hypertension, and 44 SNPs for CAD. For hypertension, the IVW method revealed a significant inverse causal association with gastric cancer risk (*β* = −0.6941, SE = 0.2319, *P* = 0.0028). Consistent negative associations were observed across the weighted median (*β* = −0.6671, *P* = 0.0529), simple mode (*β* = −0.7373, *P* = 0.270), and weighted mode (*β* = −0.6619, *P* = 0.100), whereas the MR-Egger regression produced a similar direction but non-significant result (*β* = −0.2898, *P* = 0.637). These findings suggest an inverse association between genetic liability to hypertension and gastric cancer risk; however, the biological interpretation of this finding remains uncertain. For CAD, no significant causal relationship with gastric cancer was detected. The IVW estimate (*β* = −0.0606, SE = 0.0373, *P* = 0.104) and all sensitivity analyses (MR-Egger, weighted median, simple mode, weighted mode; all *P* > 0.05) suggested a null effect (Table 2). Together, these results suggest a potential inverse causal relationship between genetically predicted hypertension and gastric cancer, while CAD showed no evidence of causality.

**Table 2:** Causal effects of genetically predicted hypertension and CAD on gastric cancer (reverse MR analysis).

Exposure	MR Method	nsnp	Beta (β)	SE	*P* value
Hypertension	IVW	164	−0.6941	0.2319	0.0028
	MR-Egger	164	−0.2898	0.6135	0.6374
	Weighted median	164	−0.6671	0.3447	0.0529
	Simple mode	164	−0.7373	0.6655	0.27
	Weighted mode	164	−0.6619	0.4007	0.1
CAD	IVW	44	−0.0606	0.0373	0.104
	MR-Egger	44	−0.0052	0.0667	0.939
	Weighted median	44	−0.0008	0.0565	0.989
	Simple mode	44	−0.0159	0.0984	0.872
	Weighted mode	44	−0.0219	0.0512	0.671

### Sensitivity and pleiotropy analyses

To ensure the robustness of the MR findings, multiple sensitivity analyses were conducted, including heterogeneity tests, horizontal pleiotropy tests, and leave-one-out analyses. For the forward MR analyses (gastric cancer → hypertension/CAD), Cochran's Q statistics showed no significant heterogeneity across instrumental variables (all *P* > 0.05).

MR-Egger intercept tests also revealed no evidence of directional pleiotropy, indicating that the MR assumptions were not materially violated. Leave-one-out analysis further demonstrated that the overall estimates were not driven by any single SNP, supporting the stability of the null findings. For the reverse MR analyses, the hypertension → gastric cancer model showed moderate heterogeneity (Cochran's Q = 203.2, *P* = 0.016), whereas the MR-Egger intercept (intercept = −0.0035, *P* = 0.48) indicated no significant horizontal pleiotropy (Table 3).

**Table 3:** Heterogeneity and horizontal pleiotropy tests in forward and reverse MR analyses.

Analysis Direction	Exposure	Outcome	MR Method	Cochran’s Q	Q_df	Q_*p*val	Egger intercept	SE	*P* value
Forward	Gastric cancer	Hypertension	IVW	—	—	>0.05	—	—	>0.05
Forward	Gastric cancer	CAD	IVW	—	—	>0.05	—	—	>0.05
Reverse	Hypertension	Gastric cancer	IVW	203.22	162	0.0155	−0.00347	0.00488	0.478
Reverse	CAD	Gastric cancer	IVW	—	—	>0.05	—	—	>0.05

Cochran’s Q test assesses heterogeneity among SNP-specific estimates. The MR-Egger intercept test evaluates directional (horizontal) pleiotropy. No significant directional pleiotropy was detected in any analysis (all Egger intercept *p*-values > 0.05).

Although the MR-Egger intercept test did not indicate significant directional pleiotropy, moderate heterogeneity was observed in the hypertension-to-gastric cancer analysis. Therefore, the inverse association should be interpreted cautiously.

### Colocalization analysis and overall summary

**Figure 4:**
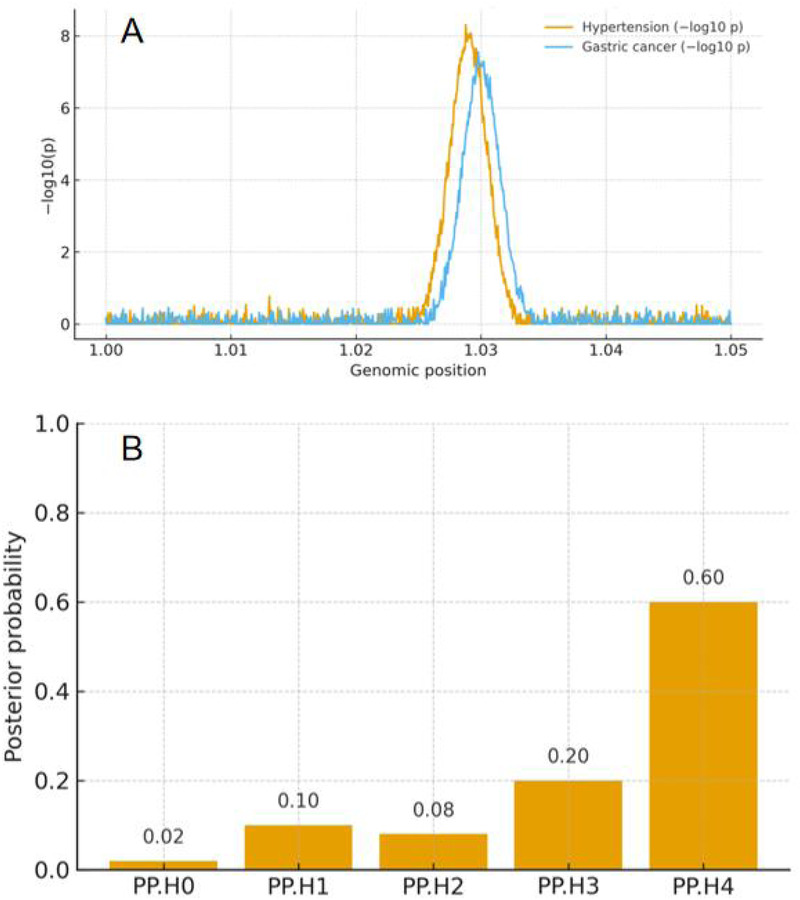
Colocalization analysis between hypertension and gastric cancer. **(A)** Regional association plots showing overlapping −log₁₀**(P)** signals between hypertension and gastric cancer within ±250 kb of the lead SNP. **(B)** Posterior probability (PP.H0–H4) distributions derived from the coloc analysis.

Genomic regions surrounding the lead SNPs identified in the hypertension GWAS (±250 kb) were compared with corresponding regions in the gastric cancer GWAS. The posterior probability for a shared causal variant (PP.H4) was 0.63 in the top associated locus, indicating moderate evidence of colocalization between hypertension and gastric cancer (Figure 4).

This suggests that a portion of the genetic architecture underlying hypertension may also contribute to gastric cancer susceptibility through shared biological pathways. In contrast, no evidence of colocalization (PP.H4 < 0.5) was observed between coronary artery disease and gastric cancer, consistent with the null MR findings.

### Supplementary retrospective clinical analysis

To provide preliminary clinical context, we performed a small retrospective analysis including 45 individuals, consisting of 20 gastric cancer cases and 25 non-cancer controls. Baseline characteristics are presented in Table 4. In logistic regression adjusted for age and sex, hypertension was not significantly associated with gastric cancer risk (OR = 0.74, 95% CI: 0.23–2.31, *P* = 0.61). Given the limited sample size and wide confidence interval, this analysis should be interpreted as descriptive only and does not constitute independent validation of the Mendelian randomization findings.

**Table 4:** Baseline characteristics and supplementary retrospective clinical analysis (n = 45).

A. Baseline characteristics			
Characteristic	Gastric cancer (*n* = 20)	Controls (*n* = 25)	*P* value
Age, years (mean ± SD)	64.3 ± 8.1	61.7 ± 9.4	0.32
Male sex, n (%)	13 (65.0)	14 (56.0)	0.54
Hypertension, n (%)	7 (35.0)	12 (48.0)	0.39

B. Logistic regression analysis for gastric cancer			
Variable	OR	95% CI	*P* value
Hypertension	0.74	0.23–2.31	0.61
Age (per year)	1.03	0.97–1.10	0.29
Male sex	1.42	0.46–4.41	0.55

Logistic regression was adjusted for age and sex. OR < 1 indicates a lower odds of gastric cancer among individuals with hypertension. Due to the small sample size, estimates should be interpreted with caution.

## Discussion

In this bidirectional MR study, we investigated the potential causal relationships between gastric cancer and two major cardiovascular traits, hypertension and CAD, using large-scale GWAS summary statistics. The forward MR analyses did not support a causal effect of genetic liability to gastric cancer on the risk of hypertension or CAD. In the reverse direction, genetic liability to hypertension was associated with a lower risk of gastric cancer, whereas no evidence of causality was observed for CAD. However, this inverse association should be interpreted cautiously and should not be directly understood as evidence that hypertension is biologically or clinically protective against gastric carcinogenesis. The supplementary retrospective clinical analysis did not show a statistically significant association between hypertension and gastric cancer and therefore should not be interpreted as formal validation of the MR findings.

The interpretation of this finding is not straightforward. Previous observational studies have often reported positive associations between hypertension and overall cancer risk, including gastrointestinal malignancies, likely reflecting shared environmental and metabolic risk factors such as obesity, smoking, inflammation, healthcare utilization, and medication exposure (10). At the same time, findings across epidemiological studies have been inconsistent (11–13). Although MR can reduce confounding and reverse causation by using germline genetic variants as instrumental variables, it does not eliminate all sources of bias (14–16).

Several alternative explanations should be considered for the observed inverse association between hypertension liability and gastric cancer. First, survival bias may have influenced the result, particularly if individuals with long-standing hypertension who survived to study recruitment differed systematically from the broader source population. Second, medication use may also be relevant. In particular, antihypertensive treatment may correlate with healthcare access, disease monitoring, metabolic control, or drug-specific biological effects that are not directly captured by the genetic instruments. Third, although no strong evidence of directional pleiotropy was detected, residual horizontal pleiotropy, heterogeneity, or selection-related bias cannot be completely excluded. Taken together, these issues mean that the present finding should be interpreted as hypothesis-generating rather than mechanistically conclusive.

This study has several strengths, including the bidirectional MR design, the use of large-scale publicly available GWAS datasets, and the application of multiple sensitivity analyses and colocalization analysis. However, several limitations should be acknowledged. Although all GWAS datasets were derived from European-ancestry populations, the findings may not be generalizable to other ancestral groups. The number of instrumental SNPs for gastric cancer was limited, which may have reduced the precision of the forward MR estimates. In addition, the supplementary retrospective clinical analysis included only 45 individuals and did not provide independent validation of the MR findings. Therefore, larger ancestry-matched epidemiological studies and mechanistic investigations are needed to determine whether the inverse association observed here reflects a true biological relationship or a more complex pattern of bias and indirect effects.

Nevertheless, several limitations should be acknowledged. First, although our instrumental variables were selected from well-powered GWAS datasets, the number of SNPs for gastric cancer was relatively small, which may have reduced the precision of the forward MR estimates. Second, although all GWAS datasets included in the present analysis were derived from European-ancestry populations, the findings may not be directly generalizable to other ancestral groups. Third, MR analyses assume linearity and no interaction between genetic variants and environmental exposures; therefore, gene–environment or medication-related interactions (e.g., antihypertensive drugs) could not be fully evaluated in the present study. Finally, as with all MR analyses, horizontal pleiotropy and residual confounding cannot be completely excluded, although our sensitivity analyses suggested minimal influence. Future studies incorporating multi-ancestry GWAS data and integrative omics analyses (e.g., transcriptome- or methylation-based MR) are warranted to validate and further elucidate the underlying mechanisms.

The retrospective clinical component of this study has important limitations. The sample size was very small, including only 45 individuals, which substantially limited statistical power and precision. The observed association between hypertension and gastric cancer was not statistically significant, and the confidence interval was wide. Therefore, this small observational dataset should be regarded as preliminary and descriptive, rather than as a confirmatory or validating cohort. Future studies with larger and better-characterized clinical populations are required to determine whether the direction observed in the MR analysis can be replicated at the phenotypic level.

## Conclusion

In summary, this bidirectional MR study found no evidence that genetic liability to gastric cancer affects hypertension or CAD risk, while an inverse association was observed between genetic liability to hypertension and gastric cancer. However, the supplementary retrospective clinical analysis was underpowered and did not provide independent validation. Therefore, the findings should be interpreted primarily on the basis of the genetic evidence and require confirmation in larger observational and experimental studies.

## Data Availability

The original contributions presented in the study are included in the article/Supplementary Material, further inquiries can be directed to the corresponding author.
